# Supplementary material for: Tandem Suzuki Polymerization/Heck Cyclization Reaction to Form Ladder-Type 9,9′-Bifluorenylidene-Based Conjugated Polymer
Source: Polymers (Basel). 2023 Aug 10;15(16):3360. doi: 10.3390/polym15163360 (PMC10458247; doi:10.3390/polym15163360)
Supplement: Supplementary file 1 [file polymers-15-03360-s001.zip › polymers-2530679-supplementary.pdf]

Electronic Supplementary Information

Tandem Suzuki Polymerization/Heck Cyclization Reaction to Form Ladder-Type 9,9′-Bifluorenylidene-Based Conjugated Polymer

Xiaoyan Zhu [1], Feng Liu [2,*], Xinwu Ba [1] and Yonggang Wu [1,*]

[1] College of Chemistry and Materials Science, Hebei University, Baoding 071002, China; zhuxiaoyan8910@126.com (X.Z.); baxw@hbu.edu.cn (X.B.)

[2] College of Basic Medicine, Hebei University, Baoding 071002, China

* Correspondence: liufeng@hbu.edu.cn (F.L.); wuyonggang@hbu.edu.cn (Y.W.)

1. Materials and measurements

The synthetic procedures were performed under argon atmosphere. Commercial chemicals (from sigma-Aldrich, JK Chemical, TCI, Soochirol Chemical Science & Technology Co., Ltd, Zhengzhou Alfa Chemical CO., Ltd and Suna Tech. Inc) were used as received. The reactions were monitored by thin layer chromatography (TLC) with silica gel 60 F254 (Merch, 0.2 mm). Column chromatography was carried out on silica gel (200-300 mesh). ^{1}H NMR and ^{13}C NMR spectra of intermedia products and monomers were recorded on a Quantum-I 400 MHz digital NMR spectrometer (Q.One Instruments Ltd., China). UV-visible absorption spectra were obtained on a Shimadzu UV-visible spectrophotometer model UV-2550. Thermogravimetric analysis (TGA) was carried out at a heating rate of 10 °C/min from 40 °C to 800 °C under a nitrogen flow using a Q50 (TA, USA). Differential scanning calorimetry (DSC) measurement was performed at the rate of 10 °C/min from -80 °C to 240 °C under a nitrogen flow using a Q2000 (TA, USA). Number average molecular weight, weight average molecular weight and polydispersity index of polymers were measured by Shimadzu LC-20AT gel permeation chromatograph (GPC) using a PLgel 5 μm Mixed-D chromatographic column and a Shimadzu RID-20A differential refractometer. Tetrahydrofuran was used as eluent at a flow rate of 1.0 mL/min at 30 °C, and universal calibration was performed with standard polystyrene samples. The electrochemical behaviores of compound and polymer were investigated by cyclic voltammetry (Holland, Ivium Plus II) with a standard three-electrode electrochemical cell in a 0.1 M tetrabutylammonium hexafluorophosphate ($TBAPF_6$) solution in CH_2Cl_2 at room temperature at atmosphere with a scanning rate of 50 mV·s^{-1}. A glassy carbon working electrode, a Pt wire counter electrode, and an $Ag/AgNO_3$ (0.01 M in CH_3CN) reference electrode were used.

2. Synthesis procedures

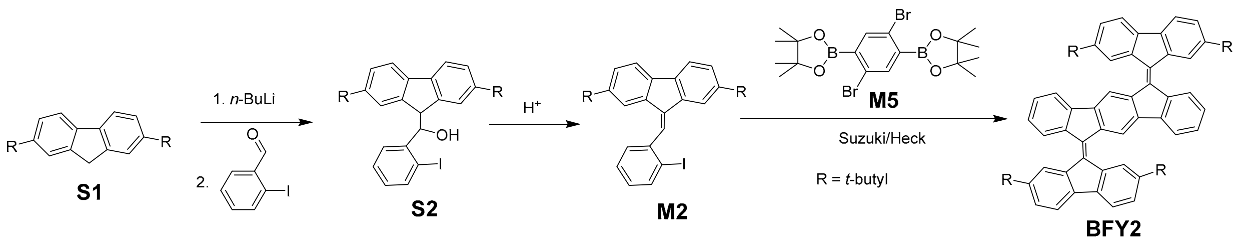

Scheme S1. Synthetic routes for **BFY2**.

Synthesis of Compound S2. Monomer S1 (0.71 g, 2.55 mmol) was dissolved in 20 mL dry THF solution. After degassing protection at a low temperature of -40 °C, n-butyl lithium (n-BuLi) (2.5 M, 1.12 mL) was added dropwise. Then the mixture was stirred at -40 °C for 0.5 h, o-iodobenzaldehyde (0.50 g, 2.15 mmol) was added slowly, the temperature of the mixture was slowly warm to room temperature. After completion of the reaction, it was extracted with water and dichloromethane. The organic phase was dried over anhydrous Na2SO4 and filtered. After removing the solvent from filtrate, the product was purified on a silica gel column with petroleum ether/dichloromethane (3:1) to obtain compound S2 as a white solid (1.07 g, 83%).

Synthesis of Compound M2. S2 (0.50 g, 0.98 mmol) and MgSO4 (0.50 g, 4.15 mmol) were dissolved into chlorobenzene (20 mL). 4-Dodecylbenzenesulfonic acid (0.64 g, 1.96 mmol) was added in the reaction mixture under argon protection. The reaction mixture was stirred at reflux for 1.5 h, and the solvent was removed by vacuum evaporation. The resulting solid was purified by column chromatography on silica gel with petroleum ether/dichloromethane (20:1) to afford compound M2 as a yellow solid (0.32 g, 67%). ^{1}H NMR ($CDCl_3$, 400 MHz): δ (ppm) 8.04 (d, *J*=8.0 Hz, 1H), 7.85 (s, 1H), 7.64-7.56 (m, 3H), 7.46 (s, 1H), 7.44-7.42 (m, 2H), 7.33 (d, *J*=8.0 Hz, 1H), 7.18 (s, 1H), 7.12 (t, *J*=7.6 Hz, 1H), 1.44 (s, 9H), 1.14 (s, 9H). ^{13}C NMR (100 MHz, $CDCl_3$) δ

(ppm) 150.0, 149.4, 141.6, 139.2, 139.0, 138.8, 137.7, 137.2, 136.7, 130.9, 129.5, 128.6, 128.0, 126.0, 125.8, 121.9, 119.1, 119.1, 117.3, 99.7, 35.1, 34.8, 31.7, 31.5, 31.3. HRMS (ESI) m/z: $[M + H]^+$ Calcd for $C_{28}H_{30}I$ 493.1392; Found 493.1396.

Synthesis of Compound M5. The reagent 1,4-Diboromo-2,5-bis(pinacolatoboryl)benzene was synthesized employing a published procedure.[S1]

Synthesis of **BFY2**. A mixture of **M2** (81 mg, 0.17 mmol), **M5** (40 mg, 0.083 mmol), $NaHCO_3$ (0.30 g), was dissolved into THF (20 mL) and H_2O (4 mL) under argon protection. After $Pd(PPh_3)_4$ (10 mg, 0.0087 mmol) was added into the mixture, the reaction mixture was stirred at reflux for 2 d. After cooling down, the reaction mixture was extracted with dichloromethane. The organic phase was dried over anhydrous Na_2SO_4 and filtered. After removing the solvent from filtrate, the crude product was purified by column chromatography on silica gel with petroleum ether/dichloromethane (10:1) to obtain pure compound **BFY2** as a purple solid (60 mg, yield 90%). ^1H NMR ($CDCl_3$, 400 MHz): δ (ppm) 8.73 (s, 2H), 8.61 (s, 2H), 8.47 (s, 2H), 8.41 (d, *J*=8.0 Hz, 2H), 7.63-7.56 (m, 6H), 7.42-7.35 (m, 4H), 7.28 (m, 2H), 7.18 (t, *J*=7.6 Hz, 2H), 1.36 (s, 18H), 1.33 (s, 18H). ^{13}C NMR (400 MHz, $CDCl_3$) δ (ppm): 149.8, 149.7, 142.3, 141.3, 140.3, 140.0, 139.3, 139.2, 139.1, 138.5, 138.4, 129.1, 126.5, 126.5, 124.6, 124.5, 119.6, 119.4, 119.3, 118.2, 35.1, 35.1, 31.6, 31.4. MS (ESI) m/z: $[M + H]^+$ Calcd for $C_{62}H_{59}$ 803.4617; Found 803.339.

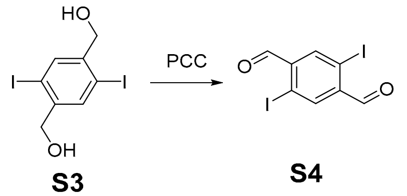

Scheme S2. Synthetic routes for **S4**.

Synthesis of Compound **S3**. The reagent (2,5-diiodo-1,4-phenylene)dimethanol was synthesized employing a published procedure.[S2]

Synthesis of Compound **S4**. The reagent 2,5-diiodoterephthaladehyde was synthesized employing a published procedure.[S3] PCC (13.66 g, 63.37 mmol) was added into a slurry of crushed molecular sieves (15.00 g) and **S3** (6.18 g, 15.85 mmol) in CH_2Cl_2 (120 mL) under argon protection. The solution was then brought to reflux for 5 h. After cooling down, the mixture was filtered through silica gel. The crude product was purified by column chromatography on silica gel with petroleum ether/dichloromethane (4:1) to obtain pure compound **S4** as a yellow solid (4.23 g, yield 69%). ^1H NMR ($CDCl_3$, 400 MHz): δ (ppm) 10.04 (s, 2H), 8.35 (s, 2H).

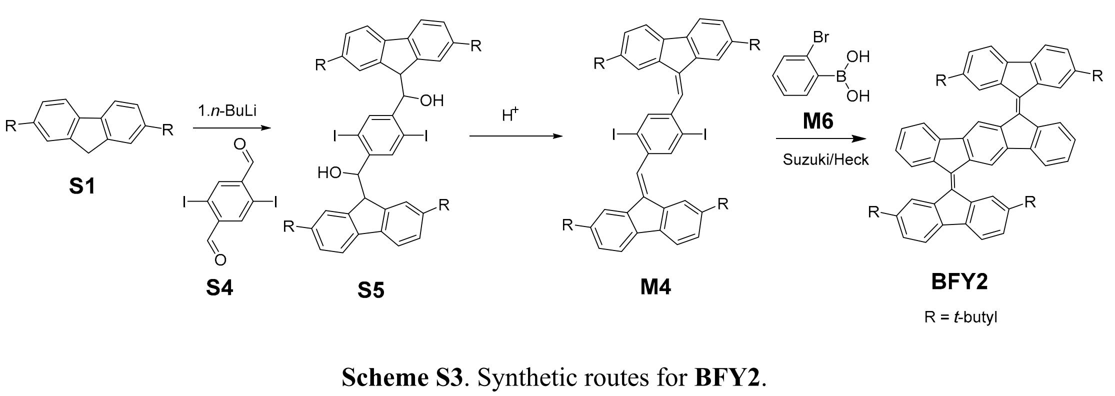

Scheme S3. Synthetic routes for **BFY2**.

Synthesis of Compound **S5**. Monomer **S1** (2.20 g, 7.9 mmol) was dissolved in 30 mL dry THF solution. After degassing protection at a low temperature of -30 °C, n-butyl lithium (n-BuLi) (2.5 M, 3.32 mL) was added dropwise. Then the mixture was stirred at -30 °C for 0.5 h, **S4** (1.00 g, 2.59 mmol) was added directly, the

mixture was allowed to slowly warm to room temperature. After the completion of the reaction, it was extracted with water and dichloromethane for several times. The organic phase was dried over anhydrous Na_2SO_4 and filtered. After removing the solvent from filtrate, the product was purified on a silica gel column with petroleum ether/dichloromethane (2:1) to obtain compound **S5** as a white solid (1.60 g, 65%).

Synthesis of Compound **M4**. **S5** (1.05 g, 1.11 mmol) and $MgSO_4$ (1.00 g, 8.3 mmol) were dissolved into chlorobenzene (30 mL). 4-Dodecylbenzenesulfonic acid (1.00 g, 3.06 mmol) was added in the reaction mixture under argon protection. The reaction mixture was stirred at reflux by heating mantle for 3 h, and the solvent was removed by vacuum evaporation. The resulting solid was purified by column chromatography on silica gel with petroleum ether/dichloromethane (15:1) to afford compound **M4** as a pale-yellow solid (0.55 g, 55%). ^{1}H NMR (400 MHz, $CDCl_3$) δ (ppm): 8.35 (s, 2H), 7.84 (s, 2H), 7.64 (s, 2H), 7.62-7.59 (m, 4H), 7.45-7.38 (m, 4H), 7.37 (s, 2H), 1.44 (s, 18H), 1.25 (s, 18H). ^{13}C NMR (100 MHz, $CDCl_3$) δ (ppm): 150.2, 149.8, 142.2, 140.8, 139.2, 138.9, 138.3, 137.1, 136.3, 126.9, 126.6, 126.3, 121.4, 119.4, 119.2, 117.4, 99.2, 35.1, 34.9, 31.7, 31.6, 31.5. HRMS (ESI) m/z: $[M + H]^+$ Calcd for $C_{50}H_{53}I_2$ 907.2237; Found 907.2246.

Synthesis of Compound **BFY2**. A mixture of **M4** (80 mg, 0.089 mmol), **M6** (40 mg, 0.199 mmol), $NaHCO_3$ (0.30 g), was dissolved into THF (20 mL) and H_2O (4 mL) under argon protection. After $Pd(PPh_3)_4$ (10 mg, 0.0087 mmol) was added into the mixture, the reaction mixture was stirred at reflux for 2 d. After cooling down, the reaction mixture was extracted with dichloromethane. The organic phase was dried over anhydrous Na_2SO_4 and filtered. After removing the solvent from filtrate, the crude product was purified by column chromatography on silica gel with petroleum ether/dichloromethane (10:1) to obtain pure compound **BFY2** as a purple solid (66 mg, 93%).

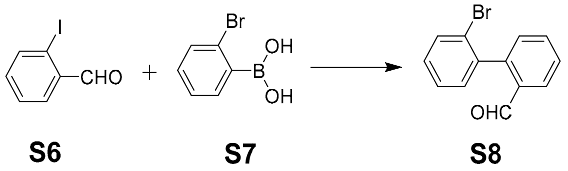

Scheme S4. Synthetic routes for **S8**.

Synthesis of Compound **S8**. A mixture of **S6** (0.50 g, 2.15 mmol), **S7** (0.43 g, 2.16 mmol), $NaHCO_3$ (0.30 g), was dissolved into THF (20 mL) and H_2O (4 mL) under argon protection. After $Pd(PPh_3)_4$ (15 mg, 0.013 mmol) was added into the mixture, the reaction mixture was stirred at reflux by microwave for 2 h. After cooling down, the reaction mixture was extracted with dichloromethane. The organic phase was dried over anhydrous Na_2SO_4 and filtered. After removing the solvent from filtrate, the crude product was purified by column chromatography on silica gel with petroleum ether/dichloromethane (3:1) to obtain pure compound **S8** as a white solid (0.53 g, 94%). ^{1}H NMR (400 MHz, $CDCl_3$) δ (ppm): 9.79 (s, 1H), 8.05 (d, *J*=7.6 Hz, 1H), 7.70-7.64 (m, 2H), 7.55 (t, *J*=7.6 Hz, 1H), 7.42 (t, *J*=7.2 Hz, 1H), 7.33-7.28 (m, 3H). ^{13}C NMR (100 MHz, $CDCl_3$) δ (ppm): 191.7, 144.5, 138.9, 133.8, 133.6, 132.8, 131.7, 130.9, 129.9, 128.7, 127.5, 127.4, 123.9. HRMS (ESI) m/z: $[M + H]^+$ Calcd for $C_{13}H_{10}BrO$ 260.9915; Found 260.9915.

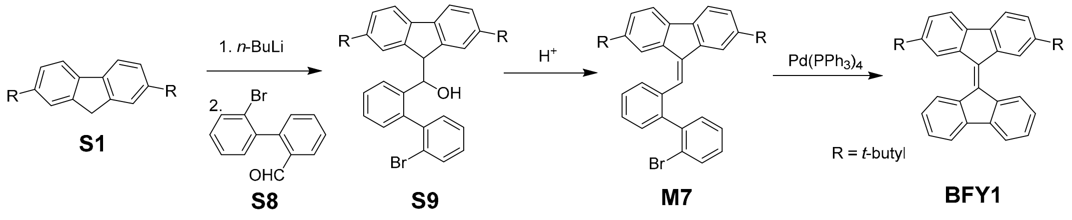

Scheme S5. Synthetic routes for **BFY1**.

Synthesis of Compound **S9**. Monomer **S1** (0.27 g, 0.96 mmol) was dissolved in 20 mL dry THF solution. After degassing protection at a low temperature of -30 °C, n-butyl lithium (n-BuLi) (2.5 M, 0.42 mL) was added dropwise. Then the mixture was stirred at -30 °C for 0.5 h, **S8** (0.20 g, 0.77 mmol) was added directly, the mixture was allowed to slowly warm to room temperature. After the completion of the reaction, it was extracted with water and dichloromethane for several times. The organic phase was dried over anhydrous Na_2SO_4 and filtered. After removing the solvent from filtrate, the product was directly used for the next step reaction.

Synthesis of Compound **M7**. **S9** and $MgSO_4$ (0.50 g, 4.15 mmol) were dissolved into chlorobenzene (15 mL). 4-Dodecylbenzenesulfonic acid (0.50 g, 1.53 mmol) was added in the reaction mixture under argon protection. The reaction mixture was stirred at reflux by heating mantle for 1.5 h, and the solvent was removed by vacuum evaporation. The resulting solid was purified by column chromatography on silica gel with petroleum ether to afford compound **M7** as a pale-yellow solid (0.35 g, 88%). ^{1}H NMR (400 MHz, $CDCl_3$) δ (ppm): 7.70-7.65 (m, 1H), 7.57-7.53 (m, 1H), 7.51-7.46 (m, 3H), 7.45-7.35 (m, 4H), 7.33-7.27 (m, 2H), 7.25-7.10 (m, 3H), 7.08-7.02 (m, 1H), 1.31-1.28 (m, 9H), 1.15-1.12 (m, 9H). ^{13}C NMR (100 MHz, $CDCl_3$) δ (ppm): 149.7, 149.3, 141.8, 141.1, 139.4, 138.8, 138.0, 137.3, 137.1, 136.5, 132.9, 131.3, 130.8, 130.5, 129.1, 128.1, 127.9, 127.3, 125.7, 125.5, 123.8, 122.4, 119.1, 119.0, 117.2, 35.0, 34.9, 31.7, 31.5, 27.1. HRMS (ESI) m/z: $[M]^+$ Calcd for $C_{34}H_{33}Br$ 520.1766; Found 520.1732.

Synthesis of Compound **BFY1**. A mixture of **M7** (33 mg, 0.063 mmol), $NaHCO_3$ (0.30 g), was dissolved into THF (20 mL) and H_2O (4 mL) under argon protection. After $Pd(PPh_3)_4$ (6 mg, 0.0052 mmol) was added into the mixture, the reaction mixture was stirred at reflux for 2 d. After cooling down, the reaction mixture was extracted with dichloromethane. The organic phase was dried over anhydrous Na_2SO_4 and filtered. After removing the solvent from filtrate, the crude product was purified by column chromatography on silica gel with petroleum ether/dichloromethane (15:1) to obtain pure compound **BFY1** as a yellow solid (27 mg, 96%). ^{1}H NMR (400 MHz, $CDCl_3$) δ (ppm): 8.46 (s, 2H), 8.44 (d, *J*=7.6 Hz, 2H), 7.75 (d, *J*=7.6 Hz, 2H), 7.58 (d, *J*=8.0 Hz, 2H), 7.34 (t, *J*=7.6 Hz, 4H), 7.22 (t, *J*=7.2 Hz, 2H), 1.33 (s, 18H). ^{13}C NMR (100 MHz, $CDCl_3$) δ (ppm): 149.6, 142.2, 141.2, 139.8, 139.1, 138.4, 138.4, 129.0, 126.7, 126.6, 126.4, 124.4, 119.9, 119.2, 35.0, 31.4. HRMS (ESI) m/z: $[M + H]^+$ Calcd for $C_{34}H_{33}$ 441.2582; Found 441.2586.

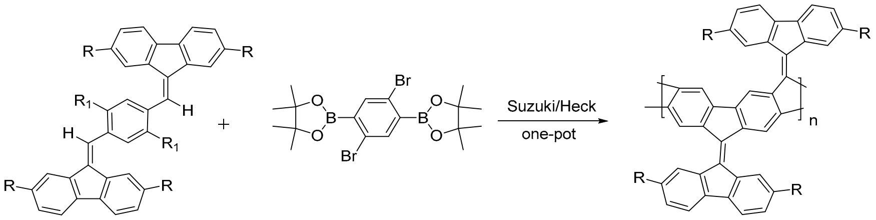

M3 (R_1=Br) **M4** (R_1=I)

M5

LCPs1 (R_1=Br) **LCPs2** (R_1=I)

R = *t*-Butyl

Scheme S6. Synthetic routes for **LCPs1** and **LCPs2**.

Synthesis of Polymer **LCPs1**. A mixture of **M3** (0.10 g, 0.12 mmol), **M5** (54 mg, 0.11 mmol), $NaHCO_3$ (0.30 g), was dissolved into THF (20 mL) and H_2O (4 mL) under argon protection. After $Pd(PPh_3)_4$ (8 mg, 0.0069 mmol) was added into the mixture, the reaction mixture was stirred at reflux for 3 d. **M1** (15 mg, 0.034 mmol) and $Pd(PPh_3)_4$ (4 mg, 0.0035 mmol) were added into the reaction mixture under argon protection. The mixture was stirred at reflux for 2 d. After cooling down, the mixture was extracted with dichloromethane. The organic phase was dried over anhydrous Na_2SO_4 and filtered. After removing the solvent from filtrate, the crude

polymer was precipitated from dichloromethane solution in methanol to give polymer **LCPs1** as deep red solid (61 mg, yield 76%). ^{1}H NMR ($CDCl_3$, 400 MHz): δ (ppm) 8.80-8.17 (broad, 3H), 7.84-7.30 (broad, 10H), 7.26-7.00 (broad, 3H), 1.56-0.85 (broad, 36H). ^{13}C NMR (100 MHz, $CDCl_3$) δ (ppm): 150.2, 149.8, 139.3, 139.0, 138.6, 137.1, 136.3, 135.5, 126.6, 126.4, 123.1, 122.7, 121.5, 119.4, 119.2, 117.5, 35.1, 34.9, 31.7, 31.5, 22.7, 14.2. Anal. calcd for $C_{56}H_{52}$: C 92.77, H 7.23; found: C 84.19, H 6.03, Br 3.26.

Synthesis of Polymer **LCPs2**. A mixture of **M4** (0.15 g, 0.17 mmol), **M5** (97 mg, 0.20 mmol), $NaHCO_3$ (0.30 g), was dissolved into THF (20 mL) and H_2O (4 mL) under argon protection. After $Pd(PPh_3)_4$ (10 mg, 0.0087 mmol) was added into the mixture, the reaction mixture was stirred at reflux for 3 d. After cooling down, **M1** (20 mg, 0.045 mmol) and $Pd(PPh_3)_4$ (4.0 mg, 0.0035 mmol) were added into the reaction mixture under argon protection. The mixture was stirred at reflux for 2 d. After cooling down, the reaction mixture was extracted with dichloromethane. The organic phase was dried over anhydrous Na_2SO_4 and filtered. After removing the solvent from filtrate, the crude polymer was precipitated from dichloromethane solution in methanol to give polymer **LCPs2** as black solid (0.11 g, yield 93%). ^{1}H NMR ($CDCl_3$, 400 MHz): δ (ppm) 8.69-8.43 (broad, 5H), 7.70-7.29 (broad, 10H), 7.20-7.18 (broad, 1H), 1.43-0.85 (broad, 36H). Anal. calcd for $C_{56}H_{52}$: C 92.77, H 7.23; found: C 85.36, H 6.46, Br 0.97.

3. GPC, DSC and TGA of the polymers

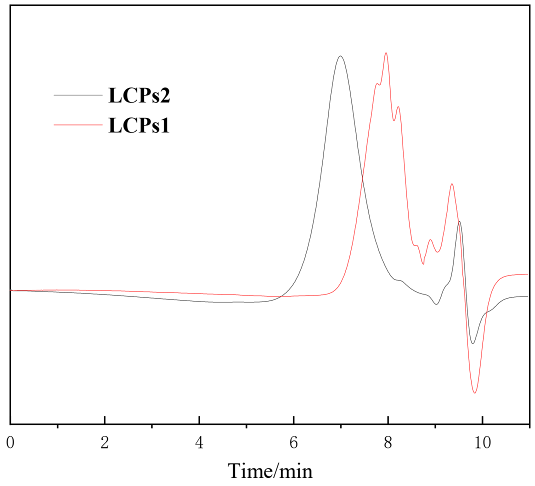

Figure S1. GPC of **LCPs1** and **LCPs2**.

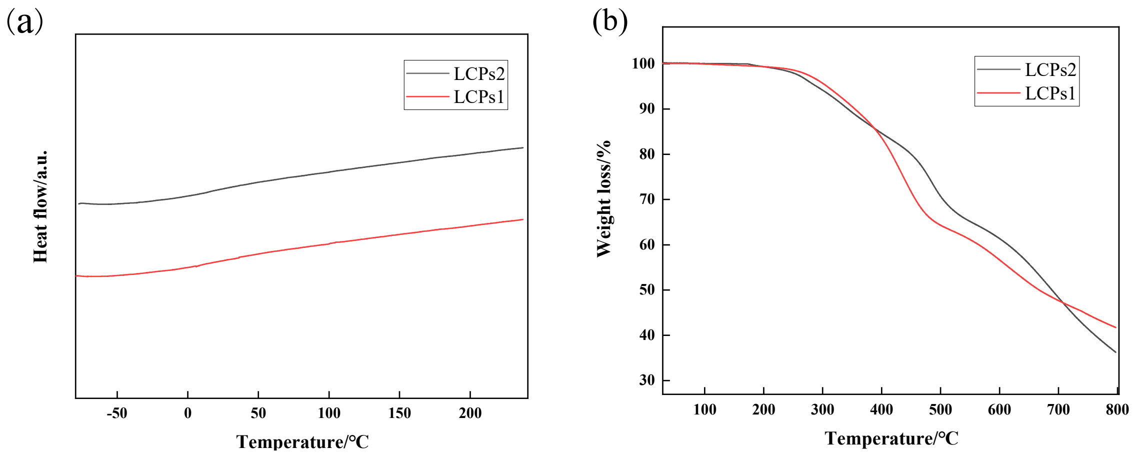

Figure S2. (a) DSC (b) TGA of **LCPs1** and **LCPs2**.

4. NMR and HRMS of the compounds and polymer

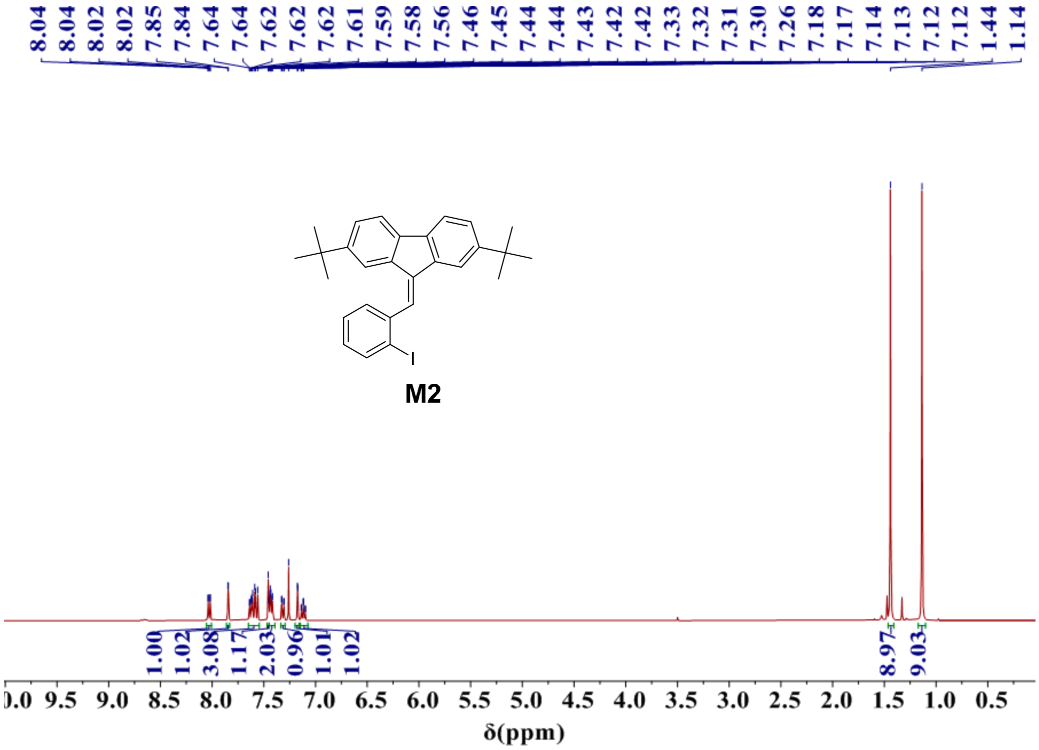

Figure S3. ^{1}H NMR of the compound **M2** recorded in $CDCl_3$.

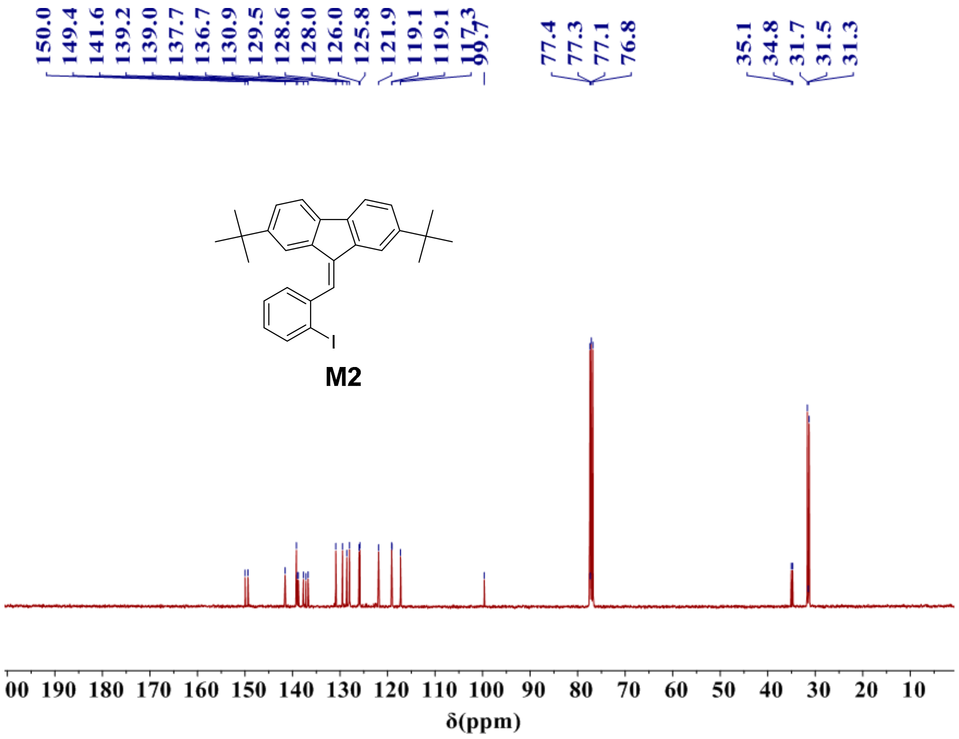

Figure S4. ^{13}C NMR of the compound **M2** recorded in $CDCl_3$.

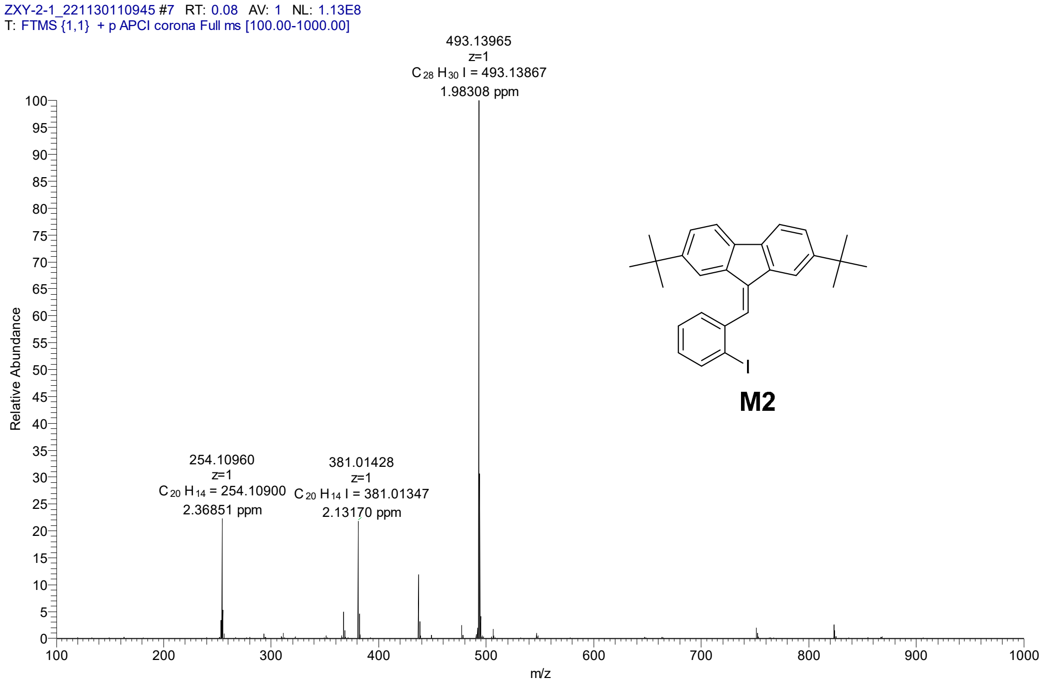

Figure S5. HRMS of the compound **M2**.

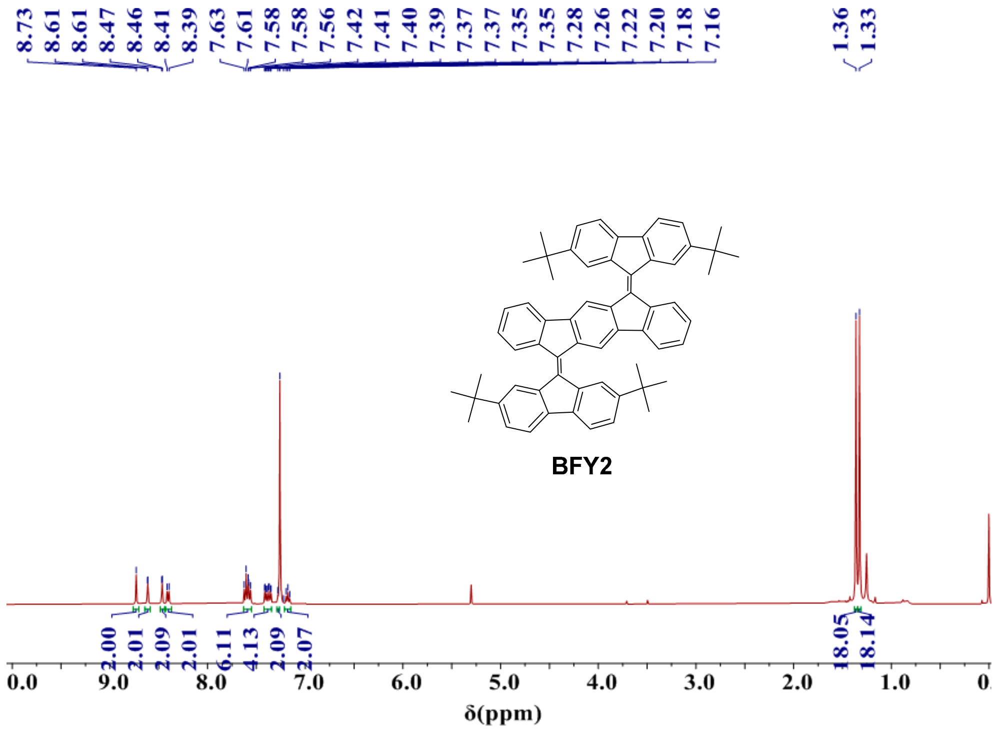

Figure S6. ^{1}H NMR of the compound **BFY2** recorded in $CDCl_3$.

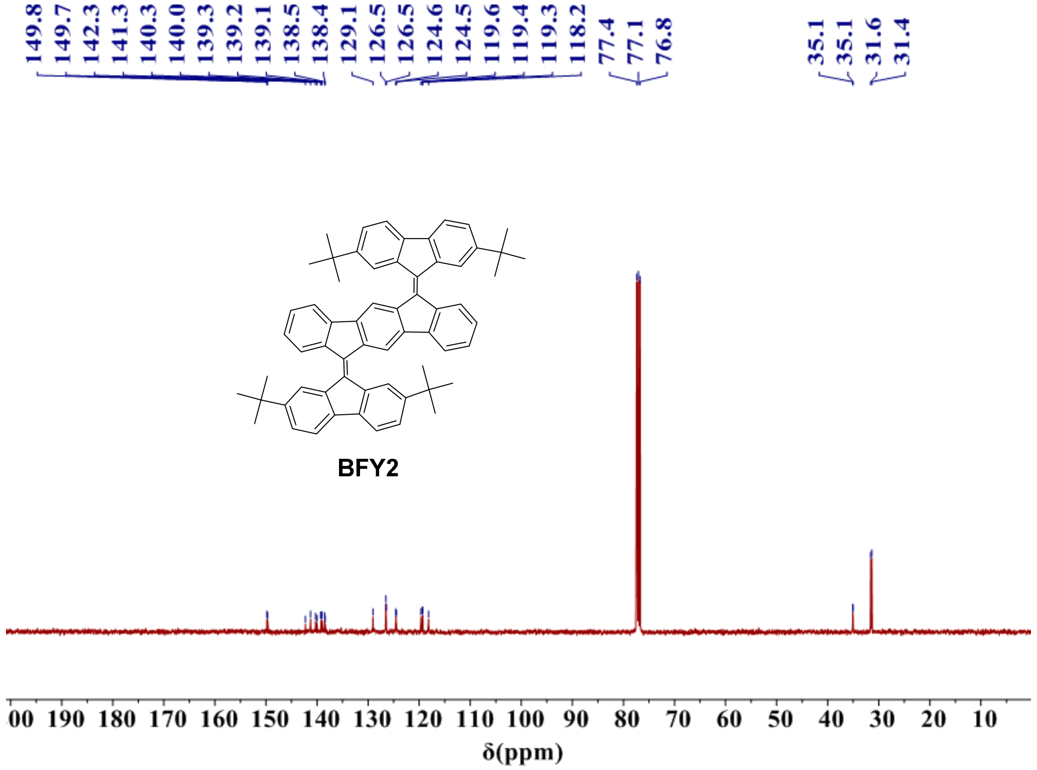

Figure S7. ^{13}C NMR of the compound **BFY2** recorded in $CDCl_3$.

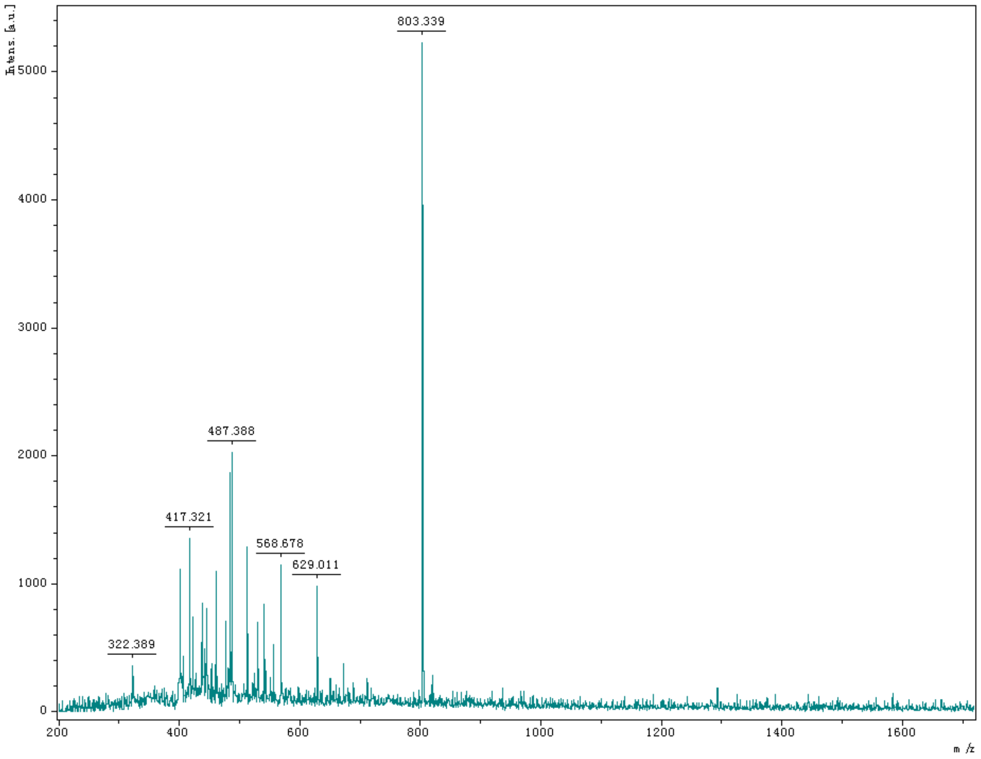

Figure S8. MS of the compound **BFY2**.

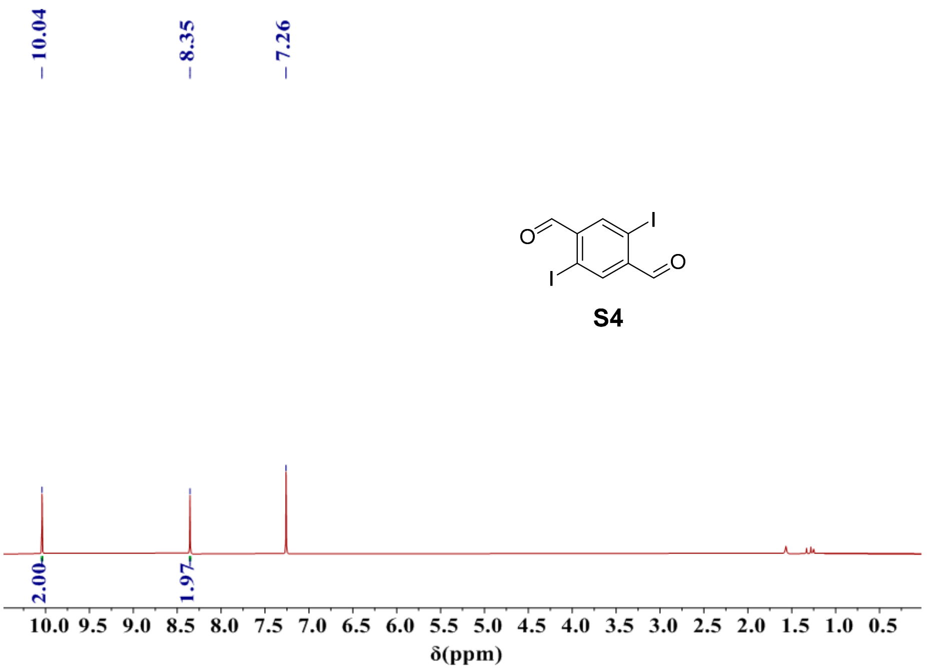

Figure S9. ^{1}H NMR of the compound **S4** recorded in $CDCl_3$.

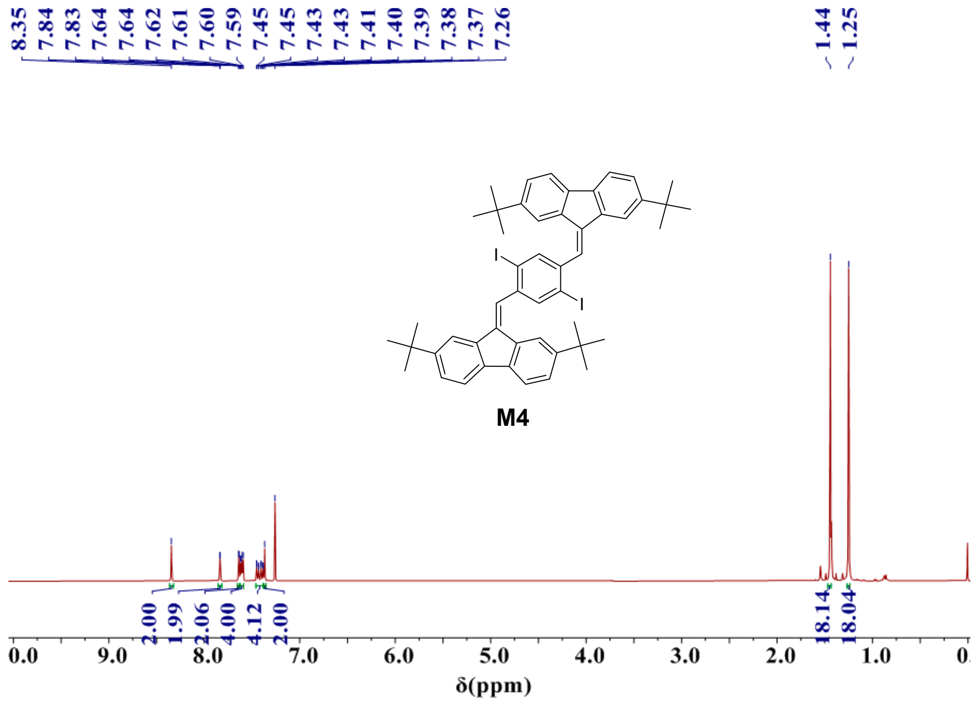

Figure S10. ^{1}H NMR of the compound **M4** recorded in $CDCl_3$.

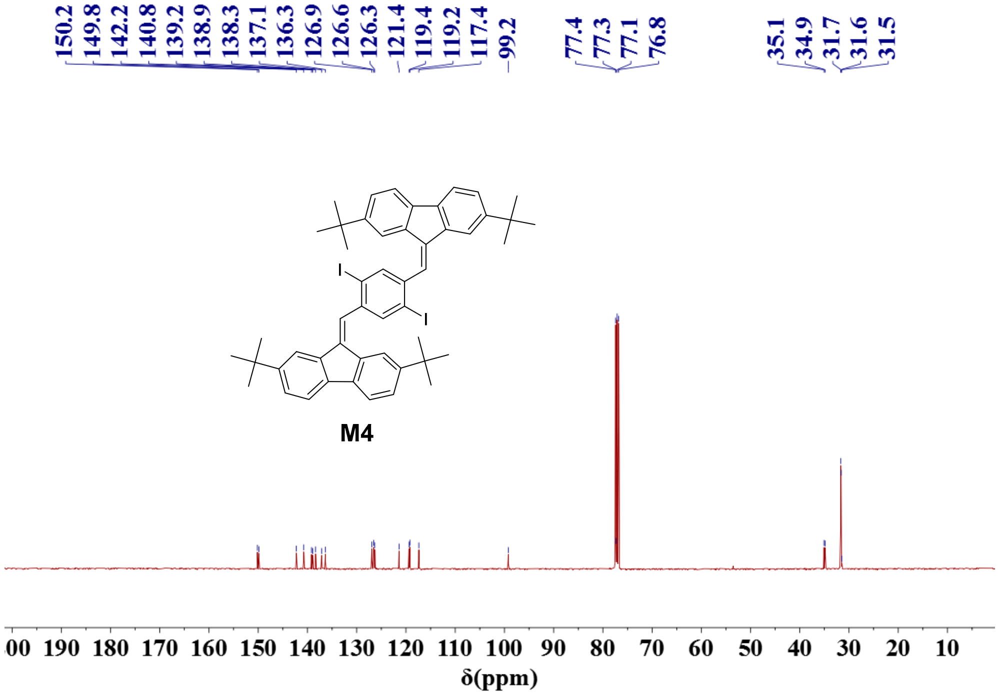

Figure S11. ^{13}C NMR of the compound **M4** recorded in $CDCl_3$.

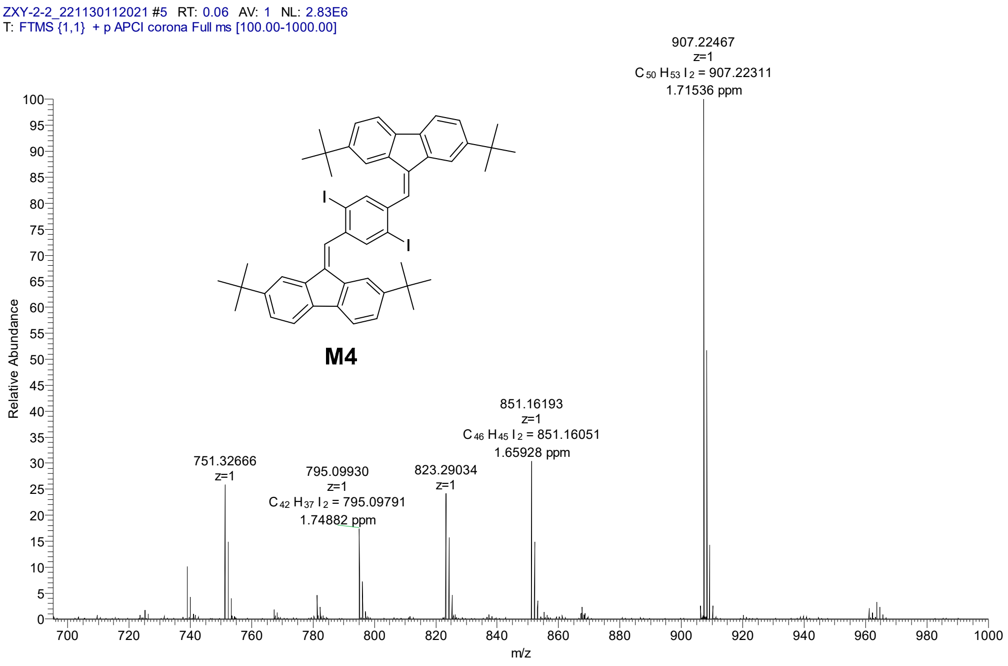

Figure S12. HRMS of the compound **M4**.

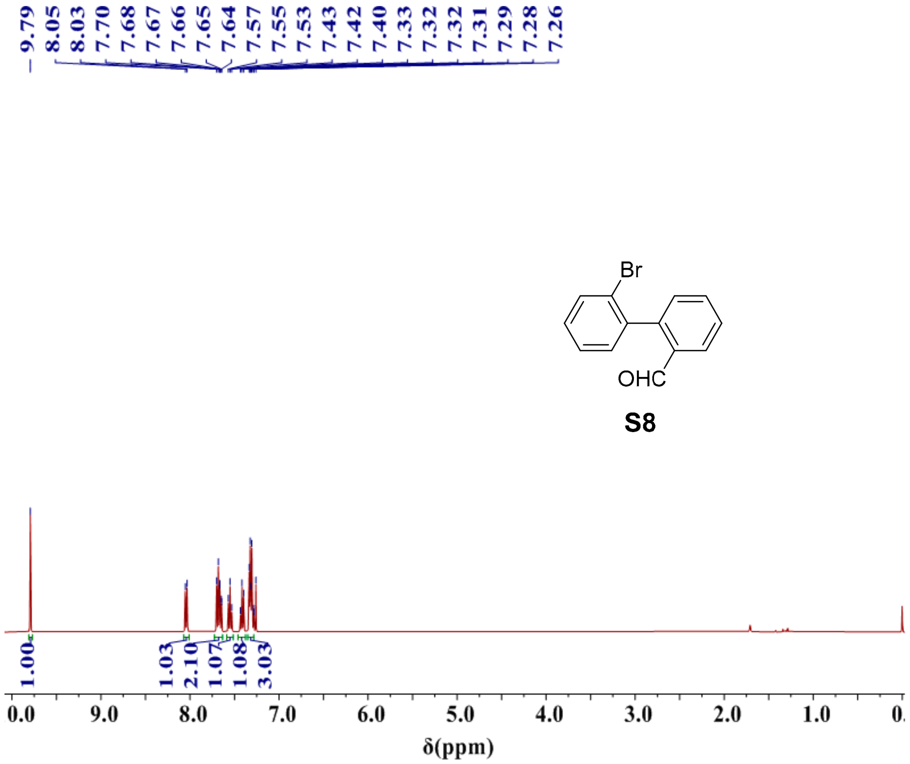

Figure S13. ^{1}H NMR of the compound **S8** recorded in $CDCl_3$.

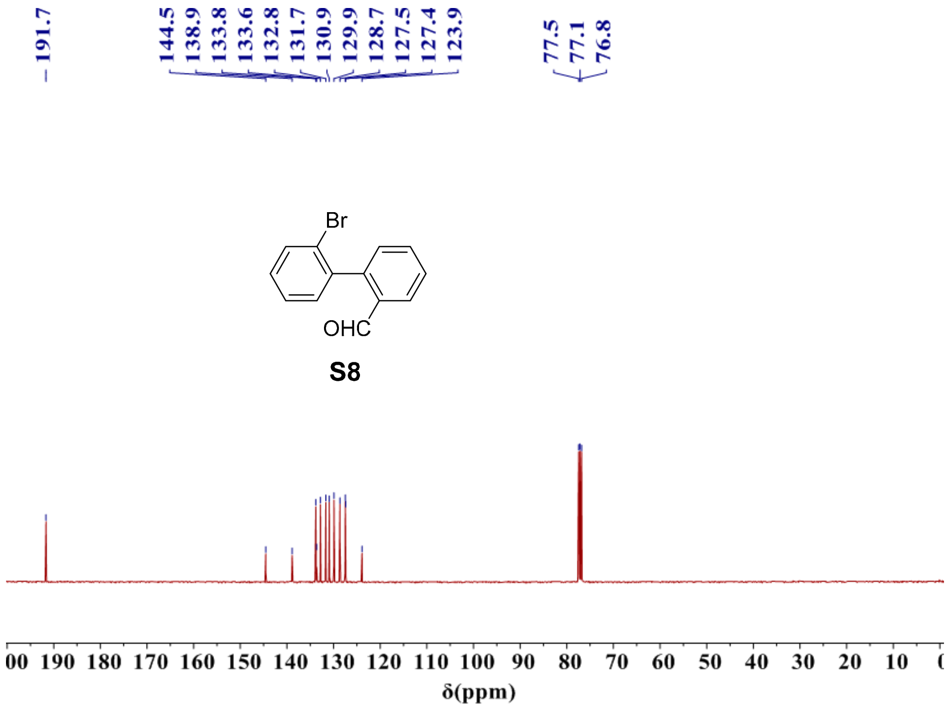

Figure S14. ^{13}C NMR of the compound **S8** recorded in $CDCl_3$.

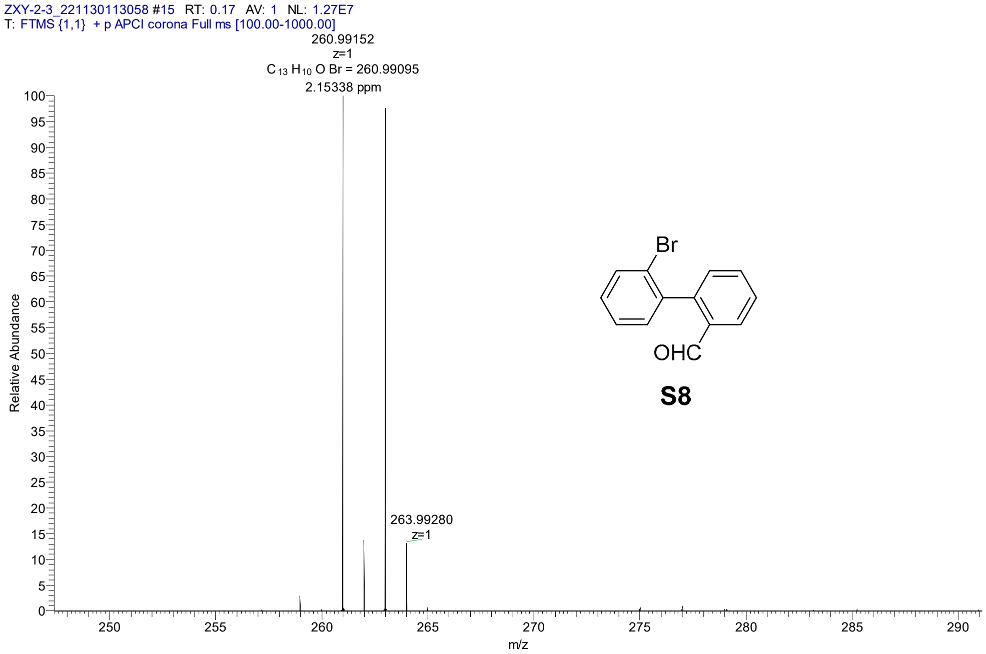

Figure S15. HRMS of the compound **S8**.

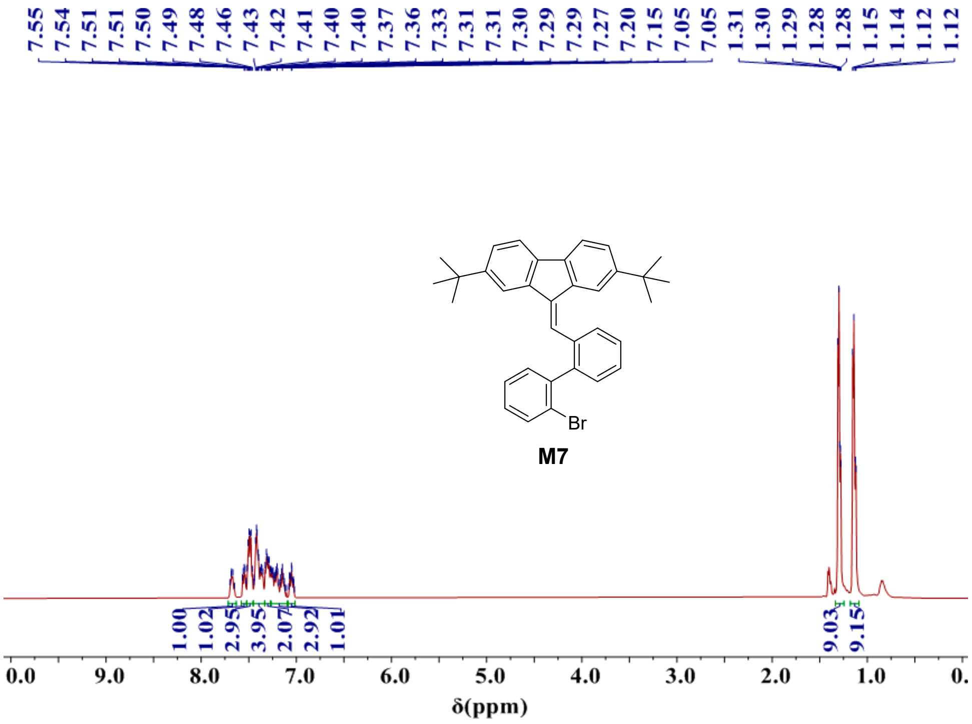

Figure S16. ^{1}H NMR of the compound **M7** recorded in $CDCl_3$.

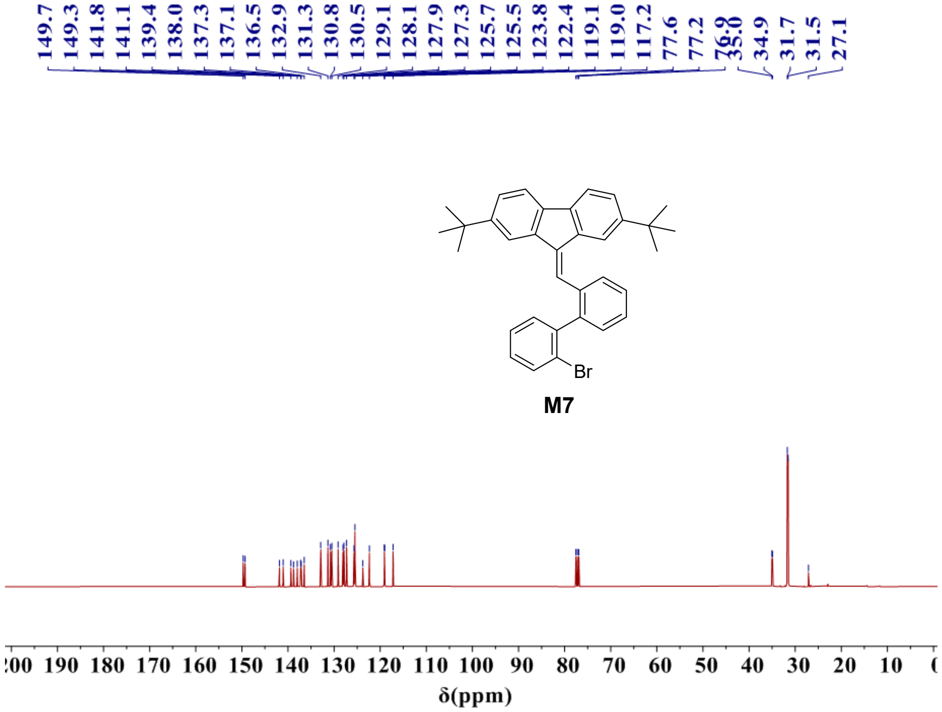

Figure S17. ^{13}C NMR of the compound **M7** recorded in $CDCl_3$.

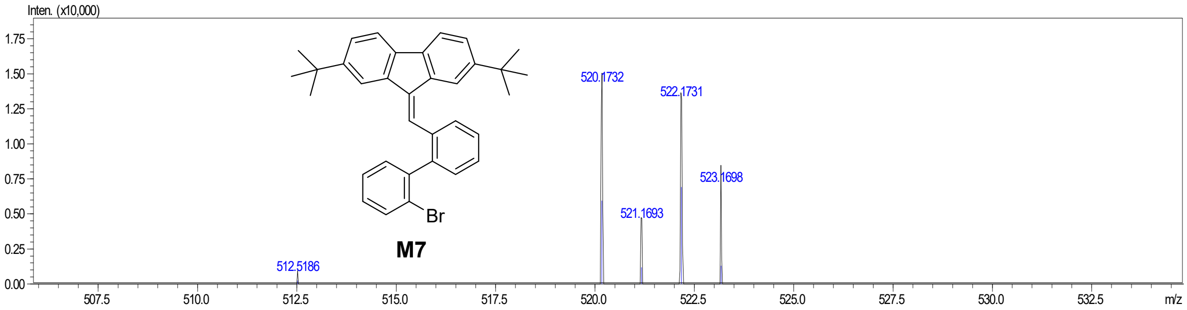

Figure S18. HRMS of the compound **M7**.

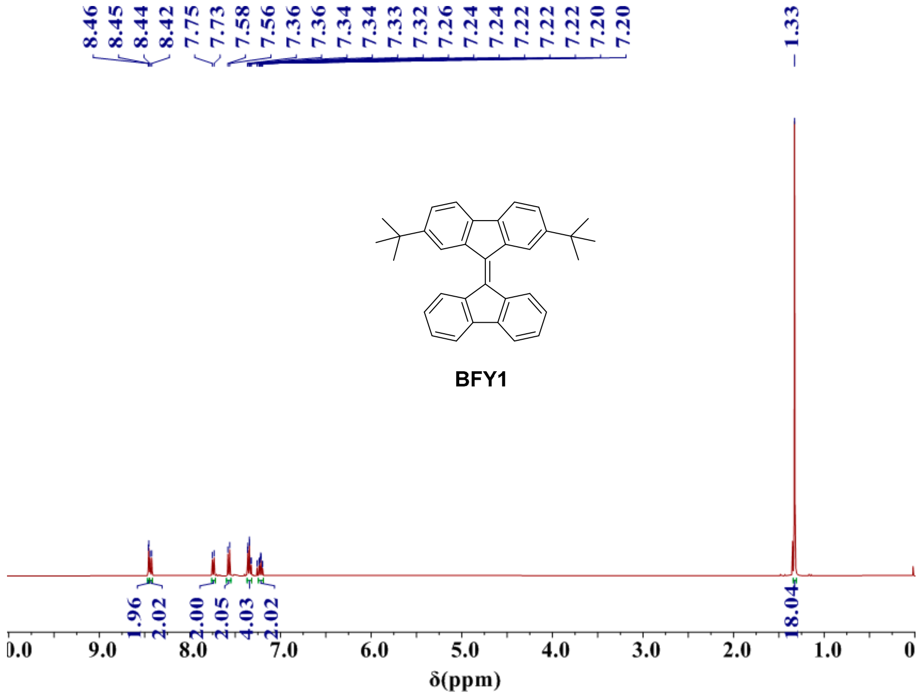

Figure S19. ^{1}H NMR of the compound **BFY1** recorded in $CDCl_3$.

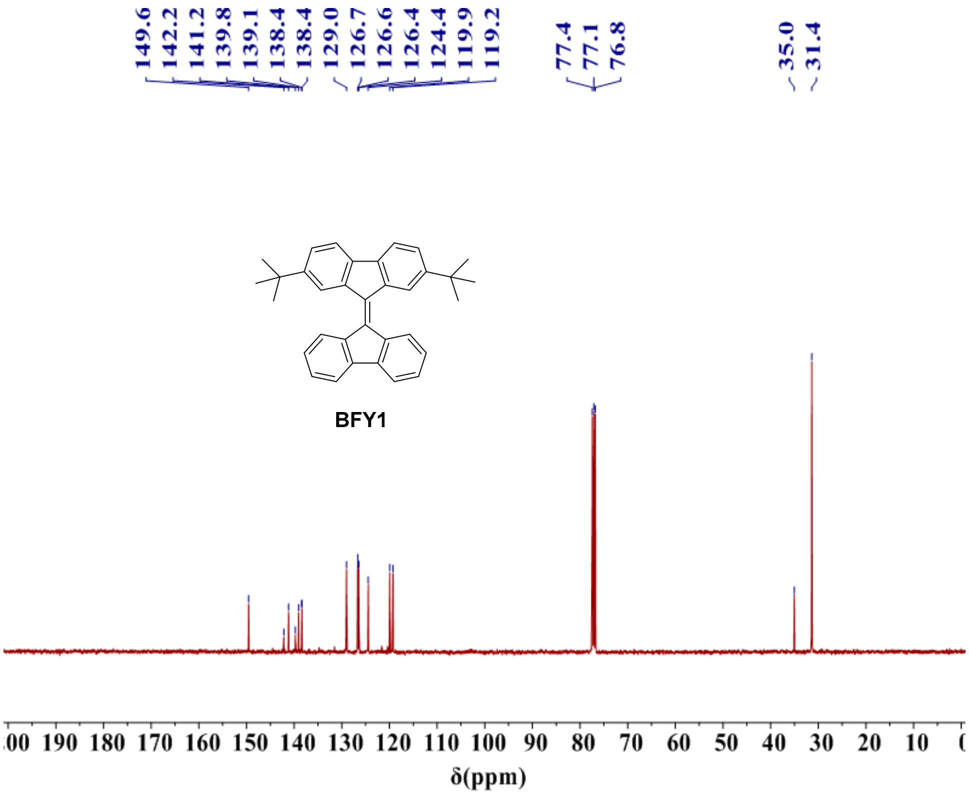

Figure S20. ^{13}C NMR of the compound **BFY1** recorded in $CDCl_3$.

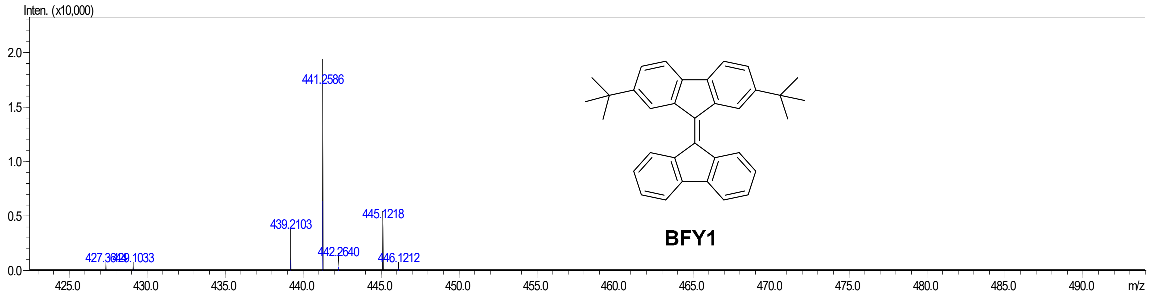

Figure S21. HRMS of the compound **BFY1**.

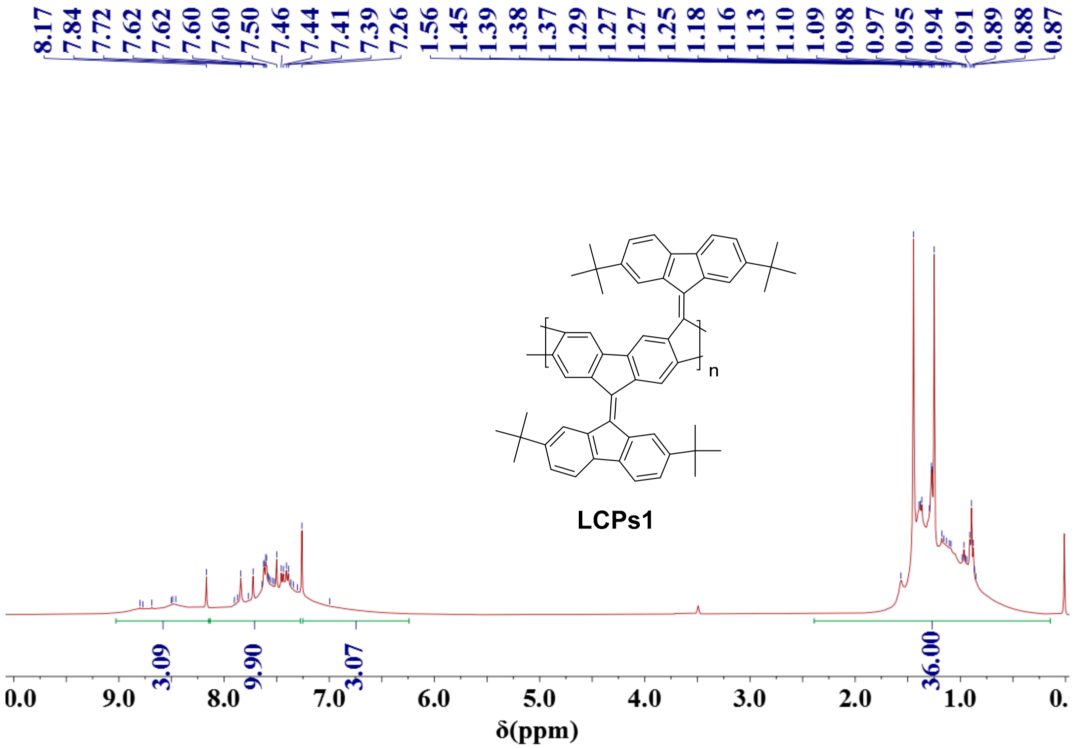

Figure S22. ^{1}H NMR of the polymer **LCPs1** recorded in $CDCl_3$.

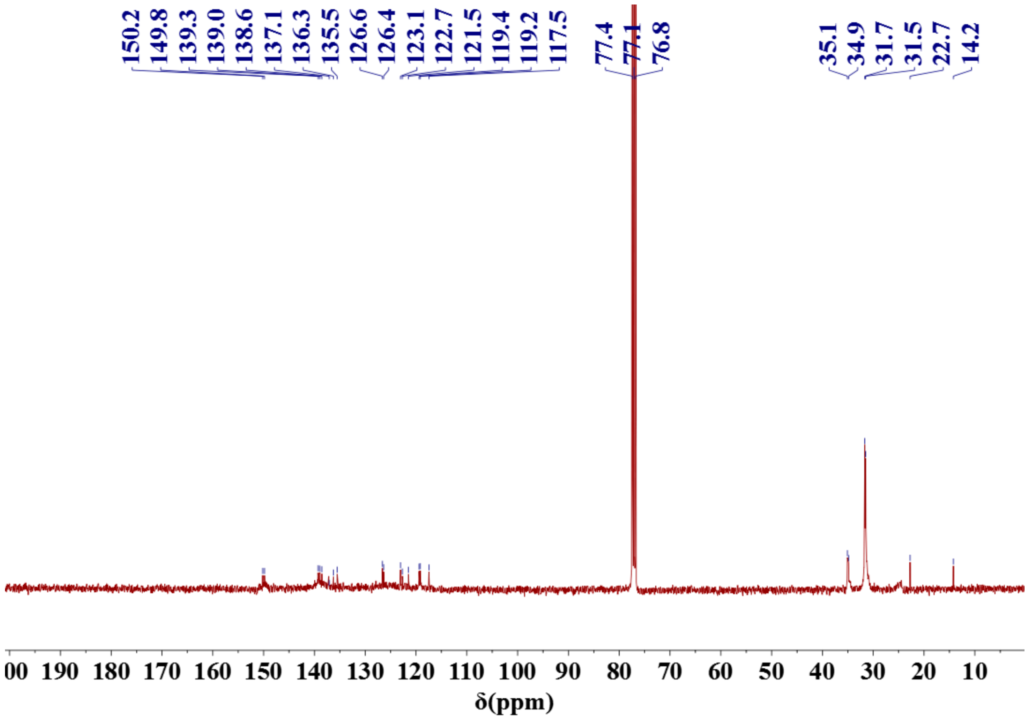

Figure S23. ^{13}C NMR of the polymer **LCPs1** recorded in $CDCl_3$.

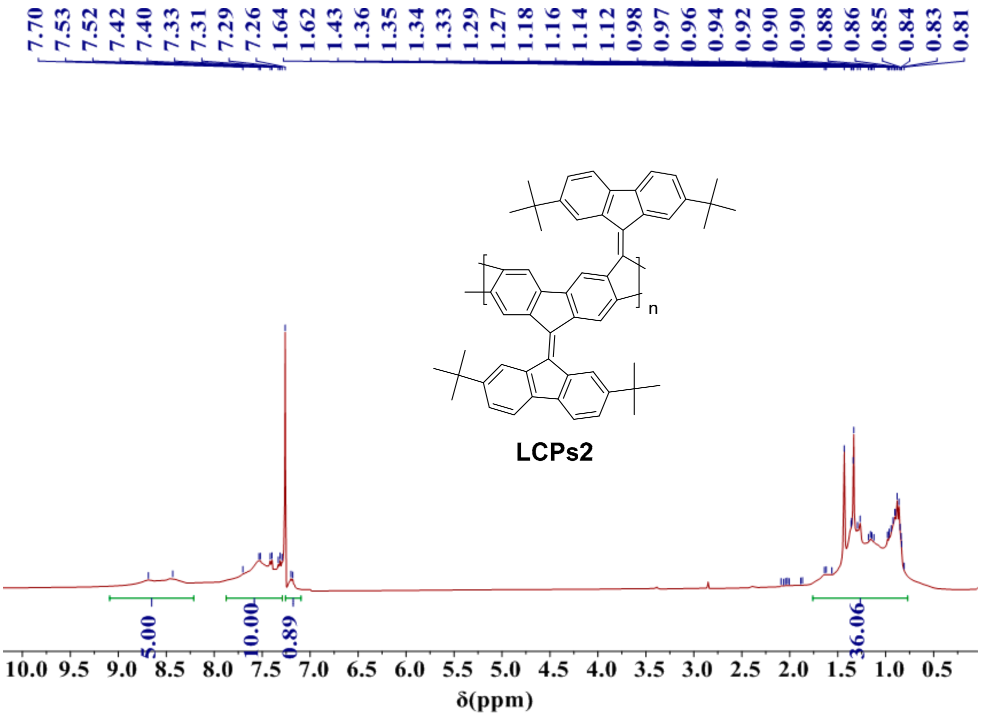

Figure S24. ^{1}H NMR of the polymer **LCPs2** recorded in $CDCl_3$.

5. References

[S1] X. Zhu, F. Liu, X. Ba, Y. Wu, *Org. Lett.* **2022**, 24, 5851.

[S2] G. Gaefke, V. Enkelmann, S. Höger, *Synthesis* **2006**, 2006, 2971.

[S3] Seo, S.; Marks, T. J. *Chemistry* **2010**, 16, 5148-5162.
